# Interpreter proxy versus healthcare interpreter for administration of patient surveys following arthroplasty: a pilot study

**DOI:** 10.1186/s12874-019-0854-1

**Published:** 2019-11-14

**Authors:** Daniel Xue, Timothy Churches, Elizabeth Armstrong, Rajat Mittal, Justine Maree Naylor, Ian Andrew Harris

**Affiliations:** 1 South Western Sydney Clinical School, Faculty of Medicine, UNSW Australia, Liverpool Hospital, Liverpool, NSW 2170 Australia; 2 Whitlam Orthopaedic Research Centre, Ingham Institute for Applied Medical Research, 1 Campbell St, Liverpool, NSW 2170 Australia; 3 South Western Sydney Local Health District, Elizabeth St, Liverpool, NSW Australia; 4 Falls, Balance and Injury Centre, Neuroscience Research Australia, 139 Barker St, Randwick, NSW 2031 Australia; 5 School of Public Health and Community Medicine, UNSW Australia, Kensington, NSW 2033 Australia

**Keywords:** Osteoarthritis, Arthroplasty, Clinical quality registry, Interpreter, Reliability

## Abstract

**Background:**

Clinical quality registries and other systems that conduct routine post-discharge surveillance of patient outcomes following surgery may have difficulty surveying patients who have limited proficiency in the language of the healthcare provider. Interpreter proxies (family and carers) are often used due to limited access to certified healthcare interpreters (due to cost or availability). The aim of this study was to assess the reliability of engaging interpreter proxies compared with certified healthcare interpreters for the administration of patient-reported health-related surveys for people with limited English proficiency (LEP).

**Methods:**

People with LEP and due for a routine 6-month telephone follow-up post knee or hip arthroplasty were invited to participate. Participants were randomly allocated to having their first interview with an interpreter proxy or a certified healthcare interpreter followed by the second (crossover) interview within 2 weeks (range: 4 to 12 days) after the first interview using the alternative method. Agreement between the two methods was assessed using quadratic weighted Cohen’s kappa, intraclass correlation and concordance correlation co-efficient where appropriate for EQ-5D health domains, total Oxford hip and knee scores, patient satisfaction, operation success, readmission, reoperation, and post-surgical complication responses. The mean of the differences between the same data items collected by each of the two methods was also calculated.

**Results:**

Eighty five participants (96%) completed the study. There was substantial to excellent inter-rater agreement (*kappa* = 0.69–0.87 and ICCs above 0.74) for all but one measure. The mean differences between family proxy and healthcare interpreter scores for each participant were small, ranging from 0.01 (score range of 1–5) to 0.72 (score range of 0–100).

**Conclusion:**

These results suggest that using interpreter proxies is a reliable alternative to certified healthcare interpreters in conducting patient-reported health surveys, potentially making this process easier and cost effective for researchers and registries.

## Introduction

Hip and knee arthroplasties are common surgical procedures and patients are commonly surveyed post-operatively to ascertain their health outcomes after treatment. A major challenge to the collection of such data is inclusion of people with limited or no language proficiency in the language of the healthcare provider or the surveyor. For those with limited English proficiency (LEP), for example, their satisfaction with healthcare may be lower, and they may have a greater risk of serious medical events [1]. In an Australian study, non-English speaking status was a predictor of lower postoperative International Knee Society scores and more severe self-reported pain following knee arthroplasty [2]. These observations suggest it is imperative that LEP patients have their health outcomes included in surveillance programs if the outcomes are to reflect the entire population.

For many outcome measurement programs, patients are surveyed by telephone. The costs of professional interpreter services [3] and logistical difficulties of finding multi-lingual staff to administer questionnaires in the patients’ native languages means that family or carers (who speak the requisite language) are often used as interpreters (termed as interpreter proxies). However, the reliability of using interpreter proxies in this setting has not been compared to the use of professional interpreters.

The aim of the study is to determine whether, in patients with LEP, the use of interpreter proxies provides adequately reliable survey results compared to using certified healthcare interpreters, which are considered the ‘gold standard’.

## Methods

We used a randomised crossover study design to compare survey outcomes between two groups: surveys conducted using interpreter proxies (family members and carers), and those conducted using certified healthcare interpreters.

The study setting was within the Arthroplasty Clinical Outcomes Registry National (ACORN), a clinical quality registry that collects health data on patients undergoing elective hip or knee arthroplasty surgery in multiple hospitals. Post-operative data collection is conducted 6 months post-surgery by telephone and approximately 12% of participants have LEP.

The participants included in this study were ACORN patients who were due for their 6-month follow-up call between March and September 2015. Inclusion criteria were: having identified themselves as requiring an interpreter and/or having LEP preoperatively, cognitive capacity to understand the follow-up questions, fluency in a language for which a healthcare interpreter is available from the South West Sydney Interpreter Services, and also having access to a family or friend proxy who is able to interpret between English and their desired language.

English proficiency was ascertained in the pre-admissions clinic through a sensitive set of questions [4]. Patients were asked *how well do you speak English?* Those who answered *very well* were deemed English proficient while those who answered *well* or *not well* were asked a second question: *In what language do you prefer your medical care?* Those nominating ‘English’ as their preferred language, were classified as English-proficient. Those nominating another language or who were unable to answer the first question were classified as having LEP and were included in the screening process for this study.

Patients with LEP who met the above criteria were sequentially ordered according to when their interviews were due and were contacted by telephone. If patients provided verbal consent with the assistance of the healthcare interpreter or interpreter proxy, they were randomly allocated to having their first interview via an interpreter proxy or via a certified healthcare interpreter. The randomisation was carried out according to a computer-generated sequence prepared before the commencement of the study and concealed in sequentially numbered envelopes containing allocation details. The envelopes were opened immediately after the patient provided consent.

The first interview was conducted within 1 month either side of the 6 month post-operative date as per routine follow up. We considered the condition to be stable at 6 months. This was followed by the second interview within 2 weeks of the first interview using the alternative method. The interview questions were asked in English by the research officer and questions and responses were translated to and from the appropriate language by the interpreter proxy or certified healthcare interpreter. Interviews with certified interpreters were made with the assistance of the call centre manager at the Sydney South West Local Health District Language Services who connected the research officer, interpreter and patient in a 3-way conference call. Interviews with interpreter proxies were performed in a 2-way telephone call with the research officer on one end and the proxy translating the interview questions and responses to and from the patient on the other end.

The questions asked were the standard 6-month follow-up questions for all patients in the ACORN registry for determining patient-reported outcome measures (PROMs) as shown below:

1. Satisfaction: “How would you describe the results of your operation” (a 5-point Likert scale - *‘excellent’*-1, *‘very good’*-2, *‘good’*-3, *‘fair’*-4, *‘poor’*-5).
2. Success: “Overall, how are the problems with your knee/hip now compared to before your operation” (a 5-point Likert scale - *‘much better’*-1, *‘a little better’*-2, *‘about the same’*-3, *‘a little worse’*-4, and *‘much worse’*-5).
3. Complications: “Have you experienced any complications after the operation since being discharged from hospital”; a standard list of common complications was read out.
4. Readmission: “Were you admitted to hospital again since leaving hospital after the knee/hip replacement?” answered as yes or no.
5. Reoperation: “Have you had another operation on the same joint that was operated on?” answered as yes or no.
6. Patient-reported health status using the EuroQoL EQ-5D-5 L and EQ-VAS questionnaires [5] (English version): The EQ-5D-5 L rates the patient’s mobility, personal care, usual activities, pain/discomfort and anxiety/depression levels in separate 5-point Likert scales, in which for each category a score of ‘1’ represents the best outcome and a score of ‘5’ the worst. The EQ-VAS rates the patient’s overall health along a visual scale from zero to 100 where zero refers to the worst health and 100 the best health. The English version of the questionnaires were used and read out by the interpreter and interpreter proxies in the patient’s desired language.
7. Joint-specific patient-reported pain and function was assessed using the Oxford Hip Score (OHS) and Oxford Knee Score (OKS) (English version). This is a 12-question survey using a Likert scale (0–4) which asks about the patient’s perceived difficulty or pain with performing everyday movements and tasks. The summary score minimum is 0 and the maximum score of 48 denotes the best outcome [6, 7]. English versions of the Oxford scores were used and read out by the interpreters and interpreter proxies in the patient’s desired language.
8. Two extra questions were asked to determine the number of years the patient and interpreter proxy had spent living in Australia: *“in what year did you (and interpreter proxy) first arrive in Australia to live here for one year or more?*” These questions correspond with those asked in the 2011 Australian Census of Population and Housing [8].

Our convenience sample exceeded the minimum sample size of 50 patients required to detect an ICC of 0.50 with 90% power and 5% significance. Ordinal data were analysed using quadratic weighted Cohen’s kappa coefficients, intraclass coefficients (ICC) measuring absolute agreement and Lin’s concordance correlation coefficient (CCC) for each outcome measure to assess the magnitude of agreement between interpreter proxy and healthcare interpreter. In addition, a Wilcoxon paired ranked sum test was performed on each measure to assess the statistical significance of the differences obtained between the two methods of interview administration. Nominal data measures were analysed with an unweighted Cohen’s kappa coefficient. The EQ-VAS was treated as continuous data and was analysed using ICC and CCC.

The differences in scores from the two methods were also determined for the EQ-5D, EQ-VAS, and Oxford scores and visualised through Bland-Altman plots [9]. From this a mean was calculated to evaluate for potential bias. The degree of bias is revealed by the mean differences and 95% Limits of Agreement plotted to indicate the range where 95% of the differences lie. All data analysis was performed using *R* open-source statistical software version 3.2 [10]. Figures were generated using R Studio version 0.99.

The outcomes assessed were the levels of agreement between the two methods of language interpreting as determined by Cohen’s *kappa* coefficients, Intraclass correlation coefficient (ICC) and Concordance correlation coefficient (CCC) statistics where appropriate. The Cohen’s kappa coefficients were interpreted in accordance with guidelines put forward by Landis and Koch [11]. Coefficients between 0.21 and 0.40 were considered to show fair agreement, scores between 0.41 and 0.60 moderate agreement, scores between 0.61 and 0.80 substantial agreement, and scores above 0.80 almost perfect agreement.

## Results

**Fig. 1:**
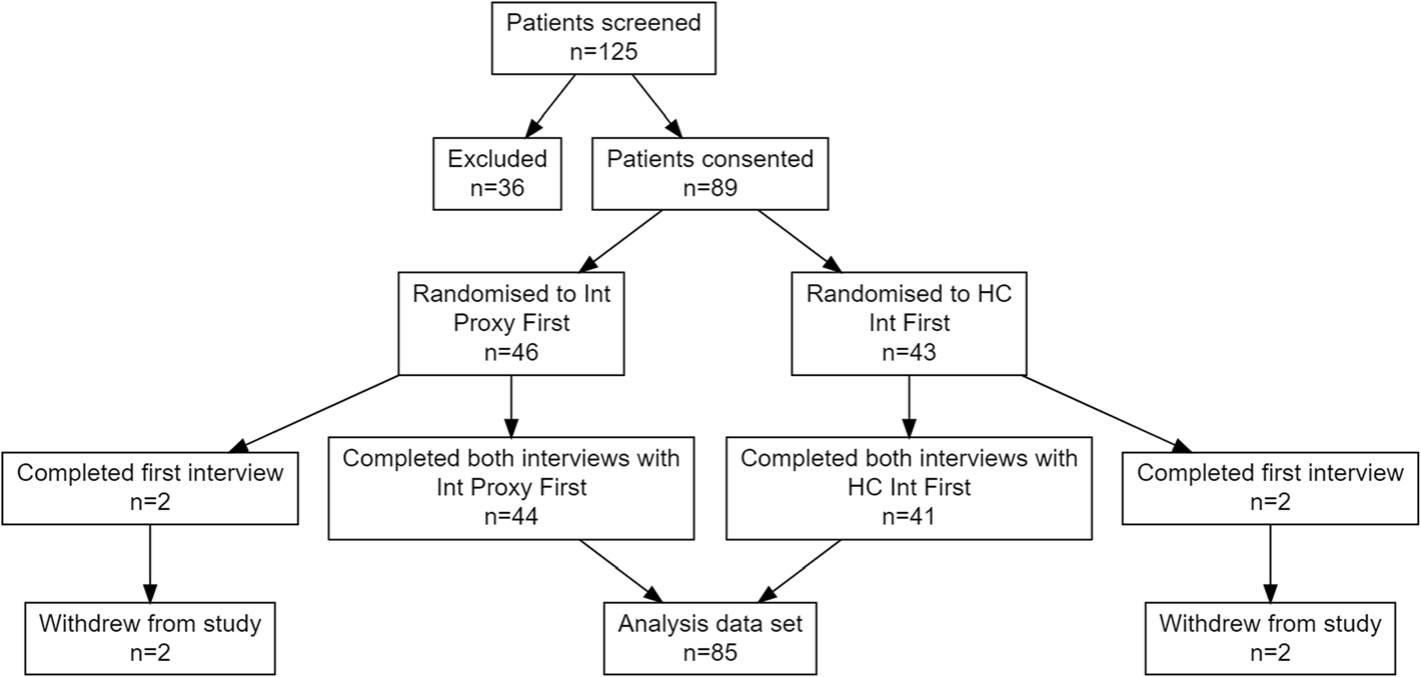
Patient flow diagram. 125 patients were identified as LEP between March 2015 and September 2015. Of these 89 were consented and randomly allocated to a call order. 46 were allocated to having the first call performed with an interpreter proxy while 43 were allocated to a healthcare interpreter first. 4 patients withdrew from the study after randomisation resulting in 85 patients being included in the final data analysis. Int Proxy: Refers to family or carers of patients acting as interpreters, HC Int: Certified healthcare interpreters

One hundred twenty-five of the patients due for follow up calls between March 2015 and September 2015 were screened and as LEP and invited to participate. Out of these, 89 patients provided consent and were given a random allocation of call order (*n* = 46, interpreter proxy first; *n* = 43, health care interpreter first). The 36 exclusions were due to not having an interpreter proxy available for translation, speaking a language for which there was no healthcare interpreter available, or if the patient was not contactable for either interview method. Four patients out of the 89 (two from each arm) withdrew from the study after consent was obtained and completion of the first interview. Eighty-five patients successfully received both methods of follow-up calls and were included in the data analysis. Figure 1 summarises the flow of patients throughout the study. Descriptive demographics were similar in both arms of the study and are displayed in Table 1 and the languages used are presented in Table 2.

**Table 1:** Demographic profile of the study population

		Total (*n*=85)	Int Proxy First (*n*=44)	HC Int First (*n*=41)
Sex, n (%)	Male	25 (29)	13 (15)	12 (14)
	Female	60 (71)	31 (36)	29 (34)
Age, mean (SD)		72 (7.7)	72 (7.1)	71 (8.5)
Patient years in Australia, mean (SD)		30 (17)	31 (16)	28 (17)
Proxy years in Australia, mean (SD)		31 (15)	32 (15)	30 (15)
Surgery Type, n (%)	Knee	66 (78)	37 (44)	29 (34)
	Hip	19 (22)	7 (8)	12 (14)

Int Proxy: Refers to family or carers of patients acting as interpreters

HC Int: Certified healthcare interpreters

*SD* standard deviation

**Table 2:** Languages utilised in this study

Language	N	Mean age	Mean years in Australia
Arabic	16	68.5	27.2
Spanish	11	76.6	33.2
Chinese	11	71.8	17.8
Greek	8	77.6	51.1
Macedonian	7	68.9	38.0
Italian	6	76.0	40.3
Serbian	4	77.5	36.2
Vietnamese	4	68.5	27.5
Assyrian	3	67.7	4.0
Punjabi	3	69.7	8.3
Croatian	2	76.5	49.0
Farsi	2	59.0	10.0
Others^a^	8	68.4	26.6

Preferred languages for the included patients and the mean number of years the patient had spent living in Australia

^a^One each for Armenian, Croatian, Haka, Maltese, Portuguese, Tamil, Turkish, Urdu

**Fig. 2:**
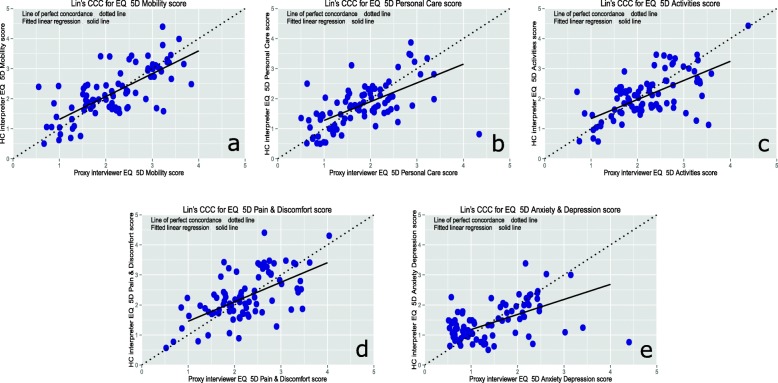
CCC plots of the separate components of EQ-5D-5L with the score from proxy interviewer plotted against healthcare interpreter: **a** mobility (CCC=0.81), **b** personal care CCC=0.66), **c** usual activities CCC=0.68), **d** pain/discomfort CCC=0.69), and (**e**) anxiety/depression CCC=0.57). A fitted linear regression (solid) is compared with a 45° line (dotted) through the origin for each plot

Agreement between methods of interview was at least substantial (agreement score > 0.60) for all outcomes except for the anxiety/depression section of EQ-5D which scored 0.57 as shown in Table 3. The remainder of the kappa scores ranged from 0.66 to 1, ICCs ranged from 0.66 to 0.87 and CCCs from 0.66 to 0.87. The CCC Plots for each domain of the EQ-5D are depicted in Table 2. Perfect agreement was seen in re-admission and re-operation responses and almost perfect agreement was seen in the Oxford score responses (*kappa and ICC =* 0.87, CCC = 0.86). Agreement for different sections of the EQ-5D varied considerably with scores from 0.57 anxiety/depression to 0.81 for mobility. The CCC Plots for each domain of the EQ-5D are depicted in Fig. 2. Patients who underwent hip arthroplasty consistently demonstrated a higher level of agreement when compared with knee arthroplasty patients.

**Table 3:** Agreement scores of patient reported outcome measures

	*kappa*	ICC (95% CI)	CCC (95% CI)	MD	95% Limits of Agreement	Wilcoxon *p*-value
EQ-5D						
Mobility	0.81	0.82 (0.73-0.88)	0.81 (0.73 - 0.87)	-0.02	-0.93 to 0.88	0.66
Personal care	0.66	0.66 (0.52-0.77)	0.66 (0.52 - 0.76)	0.02	-1.11 to 1.15	0.84
Usual Activities	0.68	0.68 (0.54-0.78)	0.68 (0.54-0.77)	0.11	-0.99 to 1.2	0.09
Pain/Discomfort	0.69	0.69 (0.56-0.79)	0.69 (0.56-0.79)	-0.02	-1.07 to 1.02	0.70
Anxiety/Depression	0.57	0.57 (0.41-0.7)	0.57 (0.41-0.69)	0.06	-1.05 to 1.16	0.38
EQ-VAS	-	0.78 (0.68-0.85)	0.78 (0.68 - 0.85)	0.72	-22.7 to 24.1	0.79
Oxford scores	0.87	0.87 (0.8-0.91)	0.86 (0.8-0.91)	0.13	-6.46 to 6.72	0.79
Satisfaction	0.75	-	-	0.01	-	0.86
Success	0.70	-	-	-0.02	-	0.71
Re-admission	1.00	-	-	-	-	-
Re-operation	1.00	-	-	-	-	-
Complications	0.69	-	-	-	-	-

Proxy: Interpreter proxy (family or carer)

HC Int: Certified healthcare interpreter

Kappa: Quadratic Weighted Cohen’s Kappa

*ICC*: Intraclass correlation

*CCC*: Concordance correlation coefficient

MD: Mean difference between the two measurements (Proxy score minus HC Int score)

95% Limits of Agreement: MD ± 1.96 SD of the MD

Score Range for EQ5D: 1-5 for each domain, EQ- VAS: 0-100, Oxford Score: 0-48, Satisfaction: 1-5, Success: 1-5

**Fig. 3:**
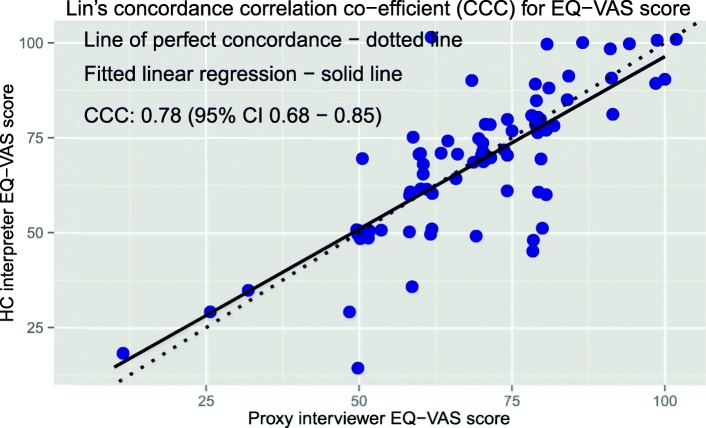
CCC plot of the EQ-VAS score (CCC=0.78)

**Fig. 4:**
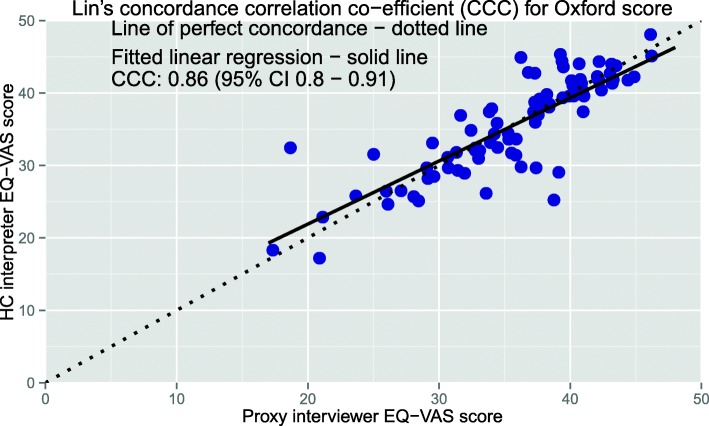
CCC plot of the Oxford hip/knee score (CCC=0.86). Data from both the hip and knee questionnaires have been analysed together

The CCC plots for the EQ VAS and Oxford scores (Figs. 3 and 4 respectively) show the scale and location shifts for the CCC analyses are minimal with the scale shifts ranging from 0.89 to 1.16 and location shifts from − 0.05 to − 0.02.

**Fig. 5:**
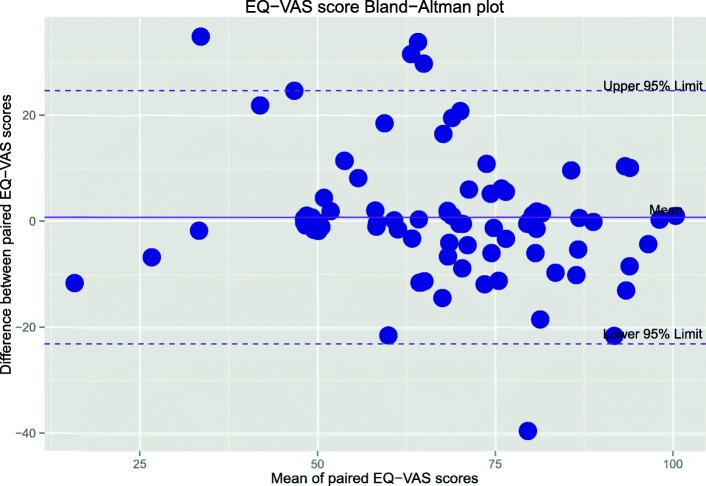
Bland-Altman plot of the EQ-VAS score. The mean of both measurements is plotted against the difference between measurements (proxy interviewer score minus healthcare interpreter score). Mean difference = 0.72 with 95% limits of agreement -22.7 to 24.1

**Fig. 6:**
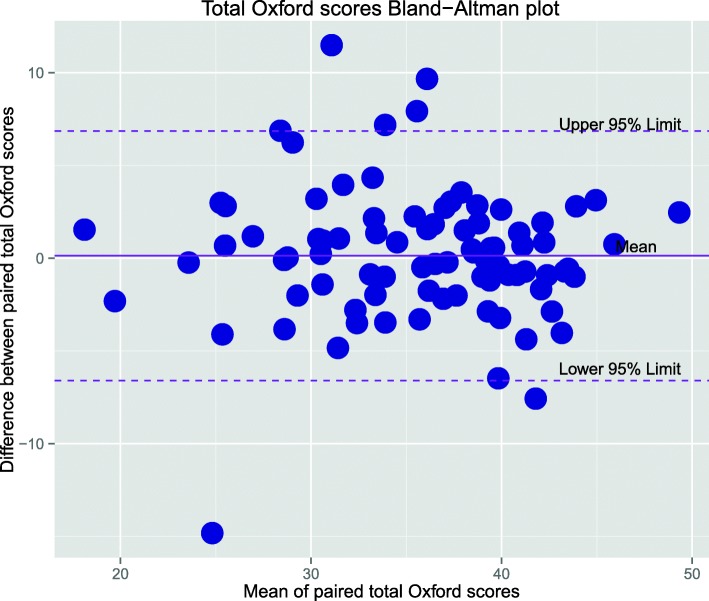
Bland-Altman plot of the Oxford hip/knee score. Mean difference = 0.13 (1.1% of total score) with 95% limits of agreement -6.46 to 6.72

The Bland-Altman plots (Figs. 5 and 6) show that relative to the total score range for each measure, the limits of agreement are narrower for the Oxford score compared with EQ-VAS. Overall the mean difference is very small (up to 1.1% of total score for the Oxford) indicating negligible bias when all subjects are considered.

The Wilcoxon rank sum tests indicated that the differences between mean scores from either method of interview were not statistically significant. When results were stratified according the interview order, the differences between mean scores remained insignificant except for the EQ-VAS mean difference where if the first call was made with a healthcare interpreter this resulted in a slightly higher score (Table 4).

**Table 4:** Mean differences stratified by interview order

	Interview Order	MD	95% Limits of Agreement	Wilcoxon *p*-value
Mobility	Proxy first	0.02	-0.87 to 0.91	0.82
	HC Interpreter first	-0.07	-0.99 to 0.85	0.35
Personal care	Proxy first	-0.02	-1.25 to 1.21	0.61
	HC Interpreter first	0.07	-0.94 to 1.09	0.41
Usual Activities	Proxy first	0.11	-1.17 to 1.40	0.31
	HC Interpreter first	0.10	-0.76 to 0.95	0.20
Pain/Discomfort	Proxy first	-0.14	-1.22 to 0.95	0.12
	HC Interpreter first	0.10	-0.86 to 1.06	0.24
Anxiety/Depression	Proxy first	0.09	-1.16 to 1.35	0.43
	HC Interpreter first	0.02	-0.90 to 0.95	0.82
EQ-VAS	Proxy first	3.73	-25.20 to 32.60	0.11
	HC Interpreter first	-2.51	-15.70 to 10.60	0.04
Oxford scores	Proxy first	0.64	-7.57 to 8.84	0.20
	HC Interpreter first	-0.42	-4.48 to 3.65	0.15

Proxy: Interpreter proxy (family or carer)

HC Int: Certified healthcare interpreter

Kappa: Quadratic Weighted Cohen’s Kappa

*ICC* Intraclass correlation

*CCC* Concordance correlation coefficient

MD: Mean difference between the two measurements (Proxy score minus HC Int score) 95% Limits of Agreement: MD ± 1.96 SD of the MD

Score Range for EQ5D: 1-5 for each domain, EQ- VAS: 0-100, Oxford Score: 0-48, Satisfaction: 1-5, Success: 1-5

## Discussion

The high level of agreement overall between interpreter proxies and healthcare interpreters found in this study suggests that utilising carers or family members to interpret is adequately reliable for capture of patient-reported outcomes after arthroplasty.

Healthcare interpreters have been noted to deliver a higher standard of interpreting quality with fewer translation errors when compared with non-certified ad hoc interpreters [12]. As such, the healthcare interpreters should be adept at clarifying any confusion over the meaning of certain words in the questionnaire and also clearly relay how the patient’s recovery profile is represented by the responses given.

The perfect agreement (*kappa* = 1) between the responses regarding readmission and reoperation was expected assuming that the question was accurately asked by both types of interpreters as these are significant events for the patient. Agreement for determining complication was lower (*kappa* = 0.69), a discrepancy which may have been due to uncertainty over the exact nature of a complication, which was defined as requiring active management but not readmission or reoperation.

The results showed that patients who had a hip arthroplasty recorded higher levels of agreement for all outcomes apart from complication, despite the only differences in questioning being the three joint-specific items in the Oxford questionnaires. This may have been due to the fact that patients who undergo knee operations experience greater day-to-day variability in their pain and function levels (possibly even dependent on weather conditions), which would account for the lower agreement levels in the EQ-5D which assesses daily situation compared with the Oxford scores which assess the most recent 4 week period.

Similar 95% limits of agreement have also been seen in test-retest studies of reliability indicating that the differences seen between methods in this study may be explained by the normal week-to-week variation of responses to the survey questions, which may also incorporate true health changes between surveys [13]. While patient recovery following arthroplasty largely plateaus after 6 months, the 1 month window either side of the 6 month date which we allowed for conducting interviews may have confounded the test-retest reliability.

However, once the entire sample population is considered, the overall bias or the mean difference on the Bland-Altman plots is consistently very small. With results stratified to interview order, there was no statistically significant difference in the mean scores except for marginal differences in the EQ-VAS score. This indicates that patients were not continuing to improve in the time between interviews. This suggests that the methods are similar when comparing groups of patients but that differences are seen at the level of the individual patient. However, as stated above, we consider this to be reflective of the reliability of the survey questions rather than due to the method of data collection.

A limitation of the study was the inability to assess the effect that individual languages may have on the level of agreement. This was due to an insufficient sample size and the large number of languages, which did not allow analysis of individual languages. While languages could potentially be grouped into geographical regions of origin, these classifications do not necessarily reflect cultural diversity and the effects this may have on patient reported outcomes. The study was limited to patients who had undergone arthroplasty surgery and these findings may not be generalisable to other settings, particularly were socially sensitive topics may be discussed which may limit the accuracy of proxy interpreters.

Another limitation is that we used English versions of the validated surveys and not those specific to the language of the patient being interviewed. Due to this, variation in the linguistic skills of proxy interpreters may have affected the accuracy of results. This approach, necessitated by a lack of availability of all required languages for each survey at the time of the study, reflected the pragmatic approach used by this registry that removes the need to restrict participation based on survey availability.

## Conclusion

The benefits of professional interpreters to the patient in a clinical setting are well recognised, with the literature agreeing that patient satisfaction and quality of care is greatest when hospital-trained interpreters and telephone interpreters are utilised [14–16]. However, the inconvenience and cost of using healthcare interpreters is a barrier to participation in data collection.

These results suggest that using interpreter proxies is a reliable, efficient and likely cost-effective alternative to using healthcare interpreters when conducting telephone surveys of patient-reported outcomes after health interventions. Despite differences seen at the individual level, when the entire cohort is considered, there is an insignificant difference between the two methods of interview.

## Data Availability

The datasets analysed during the current study are available from the corresponding author on reasonable request.

## Abbreviations

ACORN: Arthroplasty Clinical Outcomes Registry National
CCC: Concordance correlation coefficient
ICC: Intraclass correlation coefficient
Kappa: Quadratic Weighted Cohen’s Kappa
LEP: Limited English proficiency
OHS: Oxford Hip Score
OKS: Oxford Knee Score
PROMs: Patient-reported outcome measures
